# Knowledge, Practice and Barriers of Community Pharmacists Towards Asthma Management: A Cross-Sectional Study in Saudi Arabia

**DOI:** 10.3390/healthcare14091175

**Published:** 2026-04-28

**Authors:** Heba H. Salem, Ayesha Siddiqua, Refal Saeed Aljali, Ahad Ibrahim Alshardi, Refal Mansour Abusllam, Rasha Mohammed Alqahtani, Lina Saad Alshehri, Naglaa S. Bazan

**Affiliations:** 1 Department of Pharmacology, College of Pharmacy, King Khalid University, Abha 61441, Saudi Arabia; 2 Department of Clinical Pharmacy, College of Pharmacy, King Khalid University, Abha 61441, Saudi Arabia; ayshafiq@kku.edu.sa (A.S.); naglaabazan@cu.edu.eg (N.S.B.); 3 College of Pharmacy, King Khalid University, Abha 61441, Saudi Arabia; phd.refal.aljali@gmail.com (R.S.A.); ahad.alshardi@gmail.com (A.I.A.); refalabusllam@gmail.com (R.M.A.); rasha.mr4321@gmail.com (R.M.A.); linalfarhanrx@gmail.com (L.S.A.); 4 Critical Care Medicine Department, Cairo University Hospitals, Cairo University, Cairo 12613, Egypt

**Keywords:** community pharmacists, asthma, knowledge, practice, barriers

## Abstract

**Introduction**: Asthma is a chronic inflammatory disease that impairs daily functioning and quality of life. Despite effective therapies, asthma control remains suboptimal and may improve through greater engagement of community pharmacists. This study assessed the knowledge, practices, and perceived barriers of community pharmacists regarding asthma management in the Aseer region, Saudi Arabia. **Methods:** A cross-sectional study was conducted using a self-administered online questionnaire covering demographics, asthma-related knowledge, practice, and perceived barriers. **Results:** A total of 290 community pharmacists participated. Overall, 64.8% showed high asthma-related knowledge, while 51.7% reported high asthma counseling practice. Pharmacists showed strong knowledge of asthma symptoms, triggers, determinants of poor control, and counseling on medication-related adverse effects, but moderate knowledge of asthma control assessment, guideline-based management, and treatment-related side effects. Commonly reported services included patient education on asthma and medications, identification of modifiable risk factors, and discussion of treatment side effects, whereas written asthma action plans, symptom control assessment, and follow-up visits were less common. In multivariable logistic regression, high knowledge (*p* = 0.002), interest in asthma training (*p* < 0.001), and greater work experience (*p* = 0.01) were associated with higher counseling practice, while patient volume showed a borderline association (*p* = 0.051). Conversely, higher practice (*p* = 0.002), working in independent community pharmacies (*p* < 0.001), and pharmacy location (*p* = 0.034) were associated with higher asthma knowledge. **Conclusions:** Community pharmacists demonstrated moderate-to-high knowledge of asthma management, but gaps remain in guideline-based practice and follow-up. Strengthening guideline-oriented training and pharmacist integration into asthma care may improve outcomes.

## 1. Introduction

Asthma is a chronic inflammatory disease of the airways characterized by recurrent symptoms such as wheezing, breathlessness, chest tightness, and coughing, which can substantially impair daily functioning and quality of life [1]. Globally, asthma affects more than 260 million individuals and is responsible for approximately 1000 deaths each day, many of which are considered preventable through appropriate long-term management and adherence to evidence-based treatment [2,3]. Despite the availability of effective therapies, asthma control remains suboptimal in a considerable proportion of patients, largely due to poor inhaler technique, inadequate medication adherence, inconsistent follow-up, and insufficient patient education [4,5]. These deficiencies contribute to frequent exacerbations, avoidable hospitalizations, and increased healthcare costs associated with uncontrolled disease.

In Saudi Arabia, asthma represents a growing public health concern. National data indicate that approximately 14.3% of adults experience asthma symptoms [6,7]. The burden is notably higher in the Aseer region, a high-altitude area in the southern part of the country, where adult asthma prevalence has reached 19.2% [8]. Environmental and geographical factors specific to high-altitude regions, including cooler temperatures and reduced oxygen levels, may exacerbate bronchoconstriction and increase symptom severity [9,10,11]. The documented higher prevalence of asthma in Aseer compared with national averages highlights the need for region-specific healthcare workforce assessments to ensure service alignment with local epidemiological needs [7,8].

Pharmacists play an increasingly important role in the management of chronic diseases, including asthma [12,13,14,15]. Traditionally focused on medication dispensing, pharmacists, particularly those practicing in community settings, have evolved into accessible healthcare professionals who frequently serve as the first point of contact for patients seeking health advice [16,17]. Their expanded responsibilities during public health emergencies further illustrate both the potential and challenges of their evolving role [18].

The Global Initiative for Asthma (GINA) guidelines have been developed to provide up-to-date, evidence-based treatment preferences for asthma, with the aim of improving clinical outcomes [19]. Moreover, GINA guidelines emphasize a multidisciplinary approach to asthma care in which community pharmacists can play a supportive role in optimizing asthma control.

In Saudi Arabia, the transformation of pharmacy practice aligns with Saudi Vision 2030, which emphasizes strengthening primary healthcare, expanding preventive services, and optimizing healthcare professional roles to improve population health outcomes. As part of this transformation, community pharmacists are increasingly recognized as contributors to patient-centered care and chronic disease management in Saudi Arabia [20,21]. Recent Saudi evidence indicated that the expansion of pharmacists’ professional roles, including advanced clinical responsibilities, depends on addressing readiness gaps, training needs, and systemic barriers within community pharmacy practice [21,22]. These priorities are consistent with international guidance supporting the integration of pharmacists into primary healthcare teams to strengthen workforce capacity and chronic disease outcomes, including asthma [23].

Indeed, community pharmacists are well-positioned to implement asthma practice guidelines, provide medication education and counseling, reinforce adherence, assess inhaler technique, conduct follow-up monitoring, identify drug-related problems, support self-management behaviors, and facilitate early identification of poorly controlled disease in patients with asthma [15,24,25,26,27]. Moreover, studies consistently demonstrate that pharmacist-led interventions can improve asthma-related outcomes, including better asthma control and lung function as well as reduced exacerbations, emergency room visits, hospital stays, and admissions, ultimately leading to improved health-related quality of life [28,29,30,31]. To achieve these goals, pharmacists should have appropriate knowledge of the disease, medications, recent guidelines, action plans and counseling principles pertaining to asthma. However, several studies have reported persistent gaps in pharmacists’ knowledge, confidence, and practical skills related to asthma management, particularly in inhaler technique, asthma action plans, and ongoing patient follow-up [32,33,34].

In Saudi Arabia, evidence regarding community pharmacists’ involvement in asthma management remains limited and regionally variable. Although reports from central and eastern regions of Saudi Arabia have shown a positive attitude toward asthma care, deficiencies in asthma-related knowledge and counseling practices were also documented, which were often attributed to time constraints, workload, and limited training opportunities [27,35,36].

Given the high prevalence of asthma in the Aseer region and the documented asthma burden differences across Saudi regions, there is a clear need to assess pharmacists’ preparedness to contribute effectively to asthma management in this unique regional context. Despite this contextual vulnerability and the expanding role of community pharmacists within Saudi Arabia’s healthcare transformation agenda, region-specific data in this regard from the Aseer region is still lacking. Furthermore, although some studies have explored pharmacists’ roles in asthma management in Saudi Arabia, important gaps remain. Some previous investigations included mixed hospital and community samples [35], while others had a very small sample size (20–22) [27,36], thus limiting the ability to interpret and extrapolate the findings of these studies specifically within the community pharmacists’ setting across Saudi Arabia. Moreover, these studies were either qualitative [36] or relied primarily on descriptive analyses [27,35] and lacked the identification of predictors of knowledge and practice performance. Therefore, a focused, region-specific evaluation exclusively among community pharmacists of an adequate representative sample size in the Aseer region is warranted. In light of the aforementioned research gaps, the present study, for the first time in the high-prevalence region in Saudi Arabia, the Aseer region, was designed to assess community pharmacists’ knowledge regarding asthma treatment and explore asthma-related services they provide. The study also extended to identify barriers that hinder the delivery of optimal patient care by community pharmacists. Moreover, the present investigation employed multivariable binary logistic regression to identify independent predictors of knowledge and practice performance, providing more robust insights into determinants of pharmacist engagement. The findings of the current study are expected to inform targeted training programs and support the integration of community pharmacists into national strategies aimed at improving asthma control.

## 2. Methodology

### 2.1. Ethics Approval

Ethical approval for this study was granted by the Research Ethics Committee at King Khalid University under the reference number KKU-15-2025-10.

### 2.2. Study Design and Study Participants

A cross-sectional study was conducted among community pharmacists working in the Aseer region of Saudi Arabia using an online self-administered questionnaire. Pharmacists were enrolled in the study if they were licensed by the Saudi Ministry of Health and were actively practicing in community pharmacies in the Aseer region during the study period, regardless of nationality, age, or gender. Exclusion criteria included pharmacists working outside the Aseer region, unlicensed pharmacists, pharmacists employed in hospital settings, and those unwilling to participate. Participation in the study was voluntary, and the confidentiality of the pharmacists’ identities was strictly maintained.

### 2.3. Sample Size and Sampling Procedure

Raosoft online sample size calculator [37] was utilized to determine the sample size with a confidence interval of 95% and a margin of error of 5%. A response distribution of 50% was used, which represents the most conservative assumption as it assumes maximum response variability and yields the largest recommended sample size. The sample size was calculated to be 276 to represent the community pharmacist population of 976 in the Aseer region. Data were collected during May 2025 from 148 community pharmacies located in seven major cities in the Aseer region: Abha, Khamis Mushait, Namas, Ahad Rafidah, Rijal Almaa, Tanomah, and Muhayil Aseer. Data collection was accomplished by PharmD interns in the last months before their graduation, who underwent comprehensive training concerning the survey aims, components, distribution and techniques to enhance participant engagement. Following the training sessions, the interns generated a QR code for the questionnaire and disseminated it to the pharmacists during official working hours to scan it and fill in the survey online. During their visits to the pharmacies, the interns provided a clear explanation of the study’s objectives and its anticipated positive outcomes for both the pharmacists and the community. In addition, they highlighted that all responses would remain confidential and no personally identifiable information would be collected, ensuring complete anonymity. Pharmacists were informed prior to participation that completion of the survey implied informed consent. Completion of all survey items was required before submission to avoid nonresponse errors. A convenience sampling approach was used and pharmacists who were present and willing to participate during pharmacy visits were invited to complete the questionnaire. This approach was adopted due to the practical nature of recruitment during pharmacy visits and may have limited the representativeness of the sample. Although the final sample size exceeded the calculated minimum requirement (n = 290 vs. 276), the exact number of pharmacists approached could not be accurately tracked due to the distribution method; therefore, a response rate could not be reliably determined, and this approach may have introduced selection bias toward more accessible or engaged participants. The estimated number of community pharmacists in the Aseer region (n ≈ 976) was used solely for sample size determination.

### 2.4. Study Tool

A survey was developed to explore pharmacists’ knowledge, practices and barriers regarding asthma management in the Aseer region, Saudi Arabia. The questionnaire was constructed by a thorough review of relevant literature and existing guidelines, including selected elements from GINA guidelines [26,38,39,40]. Content validity of the questionnaire was assessed through expert review by five professors/assistant professors experienced in pharmacy practice, with particular attention to the relevance and clarity of the items. Based on that, the instrument demonstrated acceptable content validity. Moreover, a pilot test was conducted with a small group of 20 community pharmacists to confirm the clarity, feasibility, timing, and ease of completion. Data from the pilot phase were excluded from the final analysis.

The questionnaire consisted of four main sections. The first section gathered sociodemographic and professional characteristics, including gender, age, degree, country in which the degree was awarded, years of work experience, pharmacy location and type, average number of patients served per day, previous asthma-related training, and interest in future asthma-specific training. Asthma-related knowledge was assessed in the second section using 10 yes/no questions concerning general disease knowledge, management, counseling and side effects. Two items specifically referenced GINA guidelines to explore familiarity with international standards. Correct answers were scored as 1 and incorrect answers as 0. The individual sum of all scores was calculated, and the overall median knowledge score of participants was estimated. Participants who scored above the median value were classified as a high knowledge group, while those who scored below the median value were considered to have low knowledge. High knowledge would imply satisfactory disease symptom recognition, epidemiology and triggers, adequate understanding of guidelines and appropriateness of pharmacotherapy, correct identification of indications, as well as awareness of drug-related adverse effects and their prevention.

The third section evaluated pharmacists’ practices toward asthma care using 13 items measured on a 5-point Likert scale (always, often, sometimes, rarely, never). This section aimed to assess how frequently pharmacists were involved in asthma management in their daily practice. Responses were scored as follows: “Always” was given 5, “Often” = 4, “Sometimes” = 3, “Rarely” = 2, and “Never” = 1, and the median score of every item was calculated. The total scores for each participant were summed and the overall median practice score of all pharmacists was evaluated. Participants above the median were categorized as having high practice, and those below the median as having low practice. High practice would indicate pharmacists’ consistent provision of patient education and counseling, together with appropriate evaluation of patient needs, symptom control, adherence and therapy-related outcomes. It also includes collaborative communication with physicians to optimize treatment plans and ensure efficient patient care.

Pharmacists were categorized into high and low knowledge/practice groups using median scores as cut-off values, as the data were not normally distributed.

The final section of the survey aimed to identify various challenges that pharmacists may face when delivering asthma-related care. In this section, participants were presented with 10 potential barriers to offering different asthma care services and were instructed to select all that they had faced. The collective sum of encountered barriers by each pharmacist was estimated, and the overall median was calculated.

### 2.5. Statistical Analysis

Data were analyzed using IBM SPSS Statistics (version 30). Continuous variables were assessed for normality using histogram inspection, Q–Q plots, and the Shapiro–Wilk test. As knowledge, practice, and barrier scores showed significant departures from normality (*p* < 0.001), non-parametric statistical methods were applied. Continuous variables were summarized using the median and interquartile range (IQR), while categorical variables were described using frequencies and percentages. Group comparisons of continuous scores between two independent groups were conducted using the Mann–Whitney U test, and comparisons across more than two groups were performed using the Kruskal–Wallis test. Associations between categorical variables were assessed using the Chi-square test. Correlations between knowledge, practice, and barrier scores were examined using Spearman’s rank correlation coefficient (ρ). For multivariable analysis, only knowledge and practice scores were dichotomized into high and low categories based on their respective median values. Multivariable binary logistic regression models were constructed to identify independent predictors of high knowledge and high practice. Independent variables entered into the models included gender, country awarding the degree, years of work experience, pharmacy location, pharmacy type, number of patients served per day, previous asthma training, interest in asthma training, and the corresponding knowledge or practice variable. All predictors were entered simultaneously into the regression models using the ENTER method to control for potential confounding. Results were presented as adjusted odds ratios (ORs) with 95% confidence intervals (CIs). The internal consistency reliability of the knowledge and practice scale was assessed using Cronbach’s alpha. All statistical tests were two-tailed, and a *p*-value < 0.05 was considered statistically significant.

## 3. Results

### 3.1. Demographic Characteristics and Background of the Participating Pharmacists

A total of 290 community pharmacists agreed to participate in this study. The majority were male (60.3%). Most respondents were between 20 and 39 years of age (85.9%), and 61.4% had obtained their pharmacy degree in Saudi Arabia. Less than half of the participants (43.1%) had 1–5 years of experience. The community pharmacies where they worked were primarily located in Khamis Mushait (52.4%) and Abha (37.6%), and approximately two-thirds were chain pharmacies (67.6%). Most participants held a bachelor’s or PharmD degree, while only three had completed postgraduate studies. Additionally, 65.5% of respondents reported serving an average of 10–50 patients per day. Only 12.8% of community pharmacists had received prior training on asthma, and about 50% were interested in receiving asthma-specific training. The participants’ demographic details and background are summarized in Table 1.

### 3.2. Knowledge Scores

The median knowledge score was 7 out of 10 (IQR: 6–9). Accordingly, 188 participants (64.8%) were classified as having higher knowledge based on the median cut-off value. As shown in Table 2, community pharmacists displayed high levels of knowledge in domains related to asthma symptom recognition (90.7%), asthma triggers (84.8%), determinants of poor asthma control, including poor inhaler technique and non-adherence (90.7%), and counseling on the prevention of medication-related adverse effects (94.1%). In contrast, lower correct response rates were observed for items assessing asthma epidemiology and disease characteristics, with only 54.1% correctly identifying that the majority of asthma patients are not cigarette smokers. Knowledge related to asthma control assessment and guideline-based management, particularly regarding reliever medication use frequency and current GINA recommendations, was moderate, with correct response rates ranging from 58.3% to 60.7%. Similarly, knowledge of medication-related adverse effects, specifically that inhaled salbutamol therapy is not associated with oral candidiasis, was correct in 59.0% of respondents.

### 3.3. Practice Scores

The median practice score was 43 out of 65 (IQR: 39–46). Accordingly, 51.7% of participants were classified as having high practice, while 48.3% were in the low practice group. As shown in Table 3, the most frequently reported activities by pharmacists (median 4; IQR: 3–4) were educating patients about basic asthma facts and on medication information (indications, directions, monitoring, side effects), identifying and managing modifiable risk factors (e.g., allergens, smoking) for poor asthma outcomes, and exploring patient-reported treatment side effects. By contrast, the least frequently offered services were scheduling follow-up visits for asthma patients (median 2; IQR: 2–3). Community pharmacists displayed a moderate level of provision with regard to the remaining services.

### 3.4. Barriers to the Provision of Asthma Healthcare Services

The median barrier score was 4 (IQR: 2–5), indicating that most participants experienced multiple concurrent challenges when counselling patients with bronchial asthma. This pattern suggests that counselling difficulties are multifactorial rather than attributable to a single barrier. Barriers most often cited by the community pharmacists were the lack of time for both patients (63.1%) and pharmacists (60.7%), the incorrect patients’ perception of the pharmacists’ role (53.4%) and the absence of pharmacist-directed asthma training programs (44.5%) as well as financial incentives (43.4%) (Table 4).

### 3.5. Factors Associated with Knowledge, Practice Scores and Barriers

#### 3.5.1. Bivariate Analysis

Bivariate analysis showed no significant differences in knowledge, practice scores or perceived barriers between male and female pharmacists (all *p* > 0.05). When participants were categorized according to median scores, Chi-square analysis indicated a significant association between age group and practice level (χ^2^ = 9.63, *p* = 0.022), with pharmacists aged 40–49 years showing the highest proportion of practice scores above the median. In contrast, age was not significantly associated with knowledge (χ^2^ = 3.38, *p* = 0.336) or perceived barriers (χ^2^ = 0.89, *p* = 0.827).

Although asthma-specific training was not associated with higher knowledge scores (U = 4633.5, *p* = 0.920), trained pharmacists reported significantly higher practice scores (U = 2799.0, Z = −3.96, *p* < 0.001) and lower barrier scores (U = 2567.0, Z = −4.49, *p* < 0.001) than those who did not receive training. Likewise, pharmacists expressing interest in further asthma training did not differ in knowledge scores (U = 10,024.5, *p* = 0.491) but reported significantly higher barrier perception (U = 8500.0, Z = −2.85, *p* = 0.004) and markedly higher practice scores (U = 5794.0, Z = −6.61, *p* < 0.001).

Furthermore, knowledge scores differed significantly by country of pharmacy qualification (*p* = 0.010), with Saudi Arabia-graduated pharmacists scoring higher, while no significant differences were observed for barrier or practice scores. Nevertheless, pharmacy type was significantly associated with knowledge, practice, and perceived barriers (all *p* < 0.05), with pharmacists working in independent pharmacies having higher knowledge, whereas pharmacists from chain pharmacies reported higher practice and barrier scores. Knowledge was weakly but significantly correlated with practice (ρ = 0.207, *p* < 0.001), while perceived barriers were not significantly correlated with either knowledge or practice (Spearman correlation, *p* > 0.05). Spearman correlation analysis further showed a weak positive correlation between number of patients served per day and practice score (ρ = 0.272, *p* < 0.001), while no significant correlation was observed with knowledge score (ρ = 0.05, *p* = 0.399). All variables were subsequently included in multivariable regression models to identify independent predictors after adjustment for potential confounding. The internal consistency reliability of the practice scale was good (Cronbach’s α = 0.836), whereas the knowledge scale showed low internal consistency reliability (Cronbach’s α = 0.382).

#### 3.5.2. Multivariable Predictors of High Asthma Counseling Practice

In multivariable binary logistic regression analysis (Table 5), high knowledge level emerged as the strongest independent predictor of high asthma counseling practice (adjusted OR = 2.34, 95% CI: 1.35–4.06; *p* = 0.002). Pharmacists who expressed interest in receiving asthma-specific training were also significantly more likely to demonstrate high practice levels (adjusted OR = 2.61, 95% CI: 1.50–4.55; *p* < 0.001). In addition, greater work experience was independently associated with higher practice (adjusted OR = 1.54, 95% CI: 1.11–2.14; *p* = 0.01). A borderline association was observed for the number of patients served per day (*p* = 0.051). Other factors, including gender, prior asthma training, pharmacy type, pharmacy location, and country of qualification, were not independently associated with practice after adjustment. This indicated that practice performance was more strongly influenced by knowledge level, professional engagement, and experience than by demographic characteristics.

#### 3.5.3. Multivariable Predictors of High Asthma Knowledge

As shown in Table 6, practice level was independently associated with high asthma knowledge, with pharmacists showing high practice being more than twice as likely to exhibit high knowledge (adjusted OR = 2.38, 95% CI: 1.36–4.16; *p* = 0.002), indicating a significant association between knowledge and practice. Pharmacists working in independent community pharmacies were significantly more likely to have high knowledge compared with those working in chain pharmacies (adjusted OR = 2.99, 95% CI: 1.57–5.69; *p* < 0.001). Pharmacy location was significantly associated with asthma knowledge (*p* = 0.034). Using Abha as the reference category, pharmacists practicing in Khamis Mushait demonstrated significantly higher odds of high knowledge (adjusted OR = 4.40, 95% CI: 1.36–14.28; *p* = 0.013), whereas no significant difference was observed for other locations. Other variables, including gender, country of qualification, work experience, patient volume, and asthma-related training, were not independently associated with knowledge after adjustment.

## 4. Discussion

Community pharmacy practice in Saudi Arabia is evolving toward expanded patient-centered care in alignment with international standards and the Vision 2030 healthcare agenda [21,41]. In parallel, the growing burden of asthma highlights the need to strengthen the role of community pharmacists in chronic disease management and patient education [21,42]. Within this context, the present study provided region-specific evidence from the Aseer region, a region with higher asthma prevalence than the Saudi average, regarding community pharmacists’ knowledge, practices, and perceived barriers toward asthma management.

In the present study, community pharmacists in the Aseer region displayed strong knowledge in several foundational asthma domains, including symptom recognition, triggers, and determinants of poor asthma control. Pharmacists also showed very high awareness of counseling strategies to prevent inhaled corticosteroid–associated oral candidiasis (94.1%). However, knowledge was lower in guideline-critical management and medication-safety domains. In particular, correct identification of GINA guideline-based reliever recommendations and avoidance of SABA-only therapy was moderate when assessed against current GINA 2025 recommendations [19]. In addition, the identification of the absence of an association between inhaled salbutamol therapy and oral candidiasis was moderate, indicating a gap in adverse effect recognition despite strong knowledge of prevention counseling strategies. This pattern suggests that while pharmacists possess strong foundational asthma knowledge, familiarity with recent guideline updates may remain incomplete, highlighting the need for continuous professional education focused on updated asthma management guidelines.

When compared with earlier findings from central Saudi Arabia in 2015, the present study reported notably higher levels of asthma knowledge, particularly in domains related to pharmacotherapy and guideline-based management [35]. Importantly, the central Saudi Arabian study included both community and hospital pharmacists, rather than focusing solely on community pharmacists’ practice. That study reported substantial deficiencies in asthma pharmacotherapy and overall monitoring knowledge, despite hospital pharmacists exhibiting higher scores than community pharmacists within the same sample [35]. In contrast, the present study, conducted exclusively among community pharmacists, showed better awareness of guideline-based management concepts and counseling-related knowledge. These differences should therefore be interpreted in light of both population composition (mixed hospital and community settings versus community-only) and differences in assessment focus, as the central Saudi study placed greater emphasis on advanced pharmacotherapy and monitoring tools such as peak flow meters [35]. Collectively, these findings may indicate relatively higher current levels of asthma-related knowledge among community pharmacists in the Aseer region compared with earlier studies from different regions of Saudi Arabia, potentially reflecting increased emphasis on continuing professional development and national healthcare transformation initiatives [21,41]. However, these comparisons should be interpreted cautiously because differences in study populations, methodologies, and assessment tools may influence the observed results. Compared with studies conducted in Jordan and Egypt, pharmacists in the current study also displayed higher knowledge levels in both foundational and guideline-driven domains. The Egyptian study included a mixed population of community and hospital pharmacists, while although the Jordanian one was described as being conducted among community pharmacists, participant characteristics indicated the inclusion of a small proportion of hospital pharmacists. However, the overall knowledge findings of both studies reflected combined results rather than stratification based on practice settings. While pharmacists in Jordan and Egypt exhibited acceptable awareness of basic asthma symptoms and triggers, knowledge of updated GINA recommendations was substantially lower, particularly regarding the risks associated with SABA-only therapy and appropriate reliever selection [26,39]. Similar deficiencies in guideline awareness have been documented among pharmacists in Türkiye, where a study involving both community and hospital pharmacists reported limited familiarity with updated asthma management guidelines, despite adequate overall knowledge scores based on composite instruments [34], a pattern that is consistent with the present study.

Overall, comparison with regional studies suggests that while foundational asthma knowledge among community pharmacists is consistently strong, knowledge related to guideline-critical management and medication safety remains variable. Differences across studies appear to reflect variation in study populations, questionnaire focus, and assessment methods rather than fundamental differences in baseline knowledge. Within this context, the present study indicated comparatively higher guideline-related knowledge among community pharmacists in the Aseer region, while still identifying areas within guideline application that warrant targeted educational interventions.

Despite relatively favorable knowledge levels, only about half of the pharmacists in the present study had high asthma counseling practice. Pharmacists were most frequently engaged in educational activities, such as explaining asthma fundamentals and medication-related information, and identifying modifiable risk factors, as well as exploring patient-reported treatment side effects. Nevertheless, the more advanced practices such as assessing symptom control over the preceding four weeks, scheduling follow-up visits, and reinforcing written asthma action plans were reported less frequently. This pattern aligns closely with findings from Jordan, Ghana, and Malaysia, where pharmacists were more comfortable providing reactive counseling rather than structured follow-up and longitudinal asthma management [26,43,44]. The limited engagement in follow-up care observed in this study is particularly important when linked to national data showing that a substantial proportion of adults in Saudi Arabia have uncontrolled or partially controlled asthma. A recent systematic review by Alqahtani and his colleagues has highlighted uncontrolled asthma rates ranging from 23% to 68% across Saudi regions, with lack of education and inadequate follow-up identified as major contributors [42]. This association was further supported by patient-level evidence from the Aseer region, reporting high rates of uncontrolled asthma and suboptimal self-management practices, underscoring an unmet need for structured education and regular follow-up [45]. These findings may partly reflect the relatively limited involvement of community pharmacists in structured asthma follow-up observed in the current study. Additionally, these findings highlight the potential value of empowering community pharmacists to play a more active role in routine asthma monitoring and continuity of care through targeted guideline-oriented training and improved access to standardized asthma management tools.

The present study identified multiple co-existing barriers to asthma counseling, most notably time constraints affecting both pharmacists and patients, lack of financial incentives, insufficient training opportunities, and limited access to guidelines or asthma action plans. Pharmacists expressing interest in further asthma training in the current study might also be more aware of existing practice constraints, which could explain their higher perceived barrier scores despite stronger engagement in counseling activities. This multifactorial barrier profile mirrors findings from Malaysia, Egypt, and Jordan, where workload, lack of remuneration, and inadequate institutional support were consistently reported as key obstacles to effective asthma service delivery [26,39,44]. Importantly, pharmacists who had received asthma-specific training in this study reported significantly higher practice scores and lower perceived barriers, although training was not independently associated with knowledge or practice in the multivariable models. This pattern suggests that training may primarily support practice implementation by enhancing pharmacists’ confidence in service delivery and role engagement, rather than improving factual knowledge alone. Similar associations between training exposure and improved counseling practices without consistently contributing to higher guideline-based knowledge have been reported in studies from Jordan, Egypt, Malaysia, and Türkiye, although differences in study design and outcome measures should be considered when interpreting these findings [26,34,39,44]. Support for the impact of such training approaches was provided by pilot-tested pharmacist asthma education programs, which suggested that structured, short-duration training can improve pharmacists’ readiness to deliver asthma care services in community settings [25].

In the present study, differences by pharmacy type, where independent pharmacies’ pharmacists exhibited higher knowledge, while chain pharmacies’ pharmacists reported higher practice and barrier scores, may reflect variation in workflow structure, professional autonomy, and service expectations. Independent pharmacies tend to emphasize personalized, patient-centered services, whereas chain pharmacies often operate under more standardized, high-volume dispensing models, potentially constraining time for individualized clinical activities, as has been described in the literature on independent versus chain pharmacy practice [46]. In the multivariable analysis, pharmacy type and location emerged as significant predictors of knowledge, with pharmacists working in independent pharmacies and those practicing in Khamis Mushait demonstrating higher knowledge levels. These findings suggest that both practice setting and regional factors may influence knowledge acquisition and application.

A key strength of this study is the demonstration of a reciprocal association between knowledge and practice, where high knowledge independently predicted better asthma counseling practice, while high practice was also independently associated with higher knowledge levels. Similar reciprocal associations have been reported in Jordanian and Egyptian studies, highlighting that pharmacists who were more actively engaged in asthma care tend to show stronger alignment between knowledge and practice domains [26,39]. This interpretation is also in agreement with evidence from a systematic review of studies conducted in Arab countries, which suggested that pharmacist-provided services are more effective when pharmacists are actively integrated into patient care models [47].

Additionally, pharmacists serving a higher number of patients per day demonstrated a positive association with practice scores in bivariate analysis; however, this association was attenuated in the multivariable model, where only a borderline association was observed (*p* = 0.051), suggesting that increased patient exposure may enhance experiential learning and reinforce clinical competence, although its independent effect appears limited. However, no significant association was observed with knowledge. Similarly, greater work experience was independently associated with higher asthma counseling practice, indicating that accumulated clinical exposure and professional maturity may contribute to improved practical performance. This observation aligns with findings from Jordan and Türkiye, where pharmacists with greater patient interaction showed superior engagement in asthma care activities [26,34].

Taken together, these findings underscore the need for structured, guideline-oriented asthma training programs integrated into continuing professional development for community pharmacists in Saudi Arabia. Given the ongoing healthcare transformation under Vision 2030 and the increasing reliance on community pharmacies for chronic disease management, addressing systemic barriers such as workload, financial incentives, and access to standardized asthma care tools will be essential. This recommendation is further supported by regional and international systematic and umbrella reviews indicating that pharmacist-led asthma interventions improved asthma control, inhaler technique, medication adherence, and quality of life [31,47,48]. In addition, evidence reported that targeted training and continuing professional development (CPD) can enhance pharmacists’ asthma counseling competencies [40,49,50,51]. Furthermore, by focusing exclusively on community pharmacists and simultaneously examining knowledge, counseling practices, and perceived barriers, the present study extends previous Saudi research by providing a comprehensive evaluation of asthma management in community pharmacy practice. Moreover, unlike several earlier studies that relied primarily on descriptive analyses, the present study employed multivariable binary logistic regression to identify independent predictors of knowledge and practice performance, providing more robust insights into determinants of pharmacist engagement. Strengthening pharmacists’ roles in asthma education, follow-up, and self-management support may contribute meaningfully to improving asthma control at the population level. Within the context of Saudi Arabia’s ongoing healthcare transformation, integrating pharmacist-led asthma services into primary healthcare pathways may further strengthen chronic disease management with a special focus on the higher-prevalence regions. Achieving this integration may require supportive regulatory and policy frameworks, structured training programs, and improved access to standardized asthma care tools to enable community pharmacists to contribute effectively to patient education, monitoring, and follow-up. Although the study was conducted in a single region (Aseer), community pharmacy practice across Saudi Arabia operates under broadly similar regulatory and professional frameworks governed by national health authorities and professional regulatory bodies [21,22]. Nevertheless, regional variations in pharmacy practice models and patient populations may limit the generalizability of the findings, and multicenter studies across other regions are warranted.

## 5. Limitations

The cross-sectional design and self-reported data limit causal inference and may overestimate actual practice. As the study was conducted in a single region and did not include objective practice measures or patient outcomes, generalizability is limited. Differences in scoring approaches and cut-off definitions across studies may also affect the comparability of reported knowledge and practice levels. Additionally, the knowledge scale demonstrated low internal consistency reliability, which may be attributed to the heterogeneous nature of the items assessing different domains of asthma management, as well as the use of dichotomous (yes/no) response options without an “I don’t know” option, which may have increased guessing and reduced reliability. Because a convenience sampling approach was used, the study may also be subject to selection bias, as pharmacists who were more available or professionally engaged may have been more likely to participate. Moreover, the majority of the participants were from the two major cities in the Aseer region, which might imply potential underrepresentation of the remaining cities. The study also did not specifically address the number of asthmatic patients served by the pharmacists per day. Nevertheless, the findings provide important regional insights into community pharmacists’ roles in asthma management.

## 6. Conclusions

Community pharmacists represent an accessible and potentially important resource for supporting asthma management in Saudi Arabia. Pharmacist-led asthma interventions, including patient education, inhaler technique and medication counseling, monitoring, medication review, patient self-management and adherence support, as well as continuity of care, have been consistently associated with improved asthma outcomes. In the present study, community pharmacists in the Aseer region exhibited moderate-to-high knowledge of asthma management; however, notable gaps remain in guideline-driven practice and structured patient follow-up. In addition, pharmacists reported several barriers that may limit the delivery of comprehensive asthma care services in community pharmacy settings. Strengthening pharmacist-led interventions through targeted guideline-oriented training, improved access to asthma management guidelines and tools, and supportive health-system integration may further enhance pharmacists’ roles in asthma management. Expanding the involvement of community pharmacists in structured asthma follow-up and guideline-based care and addressing identified barriers could contribute to improving asthma control and management in Saudi Arabia.

## Figures and Tables

**Table 1:** Baseline demographics and background of the participating pharmacists (N = 290).

Characteristics	n (%)
**Gender**	
Male	175 (60.3)
Female	115 (39.7)
**Age**	
20–29	120 (41.4)
30–39	129 (44.5)
40–49	31 (10.7)
50–59	10 (3.4)
**Country Awarding the Bachelor’s/PharmD Pharmacy Degree**	
Saudi Arabia	178 (61.4)
Outside Saudi Arabia	112 (38.6)
**Location of the Community Pharmacy**	
Abha	109 (37.6)
Khamis Mushait	152 (52.4)
Other (Namas, Ahad Rafidah, Rijal Almaa, Tanomah, Muhayil Aseer)	29 (10)
**Education Level**	
Bachelor’s degree	133 (45.9)
PharmD	154 (53.1)
Master’s/Other postgraduate degree	3 (1)
**Work Experience as Community Pharmacist, years**	
<1	31 (10.7)
1–5	125 (43.1)
6–10	93 (32.1)
11–15	30 (10.3)
16–20	5 (1.7)
>20	6 (2.1)
**Type of Community Pharmacy**	
Chain Community Pharmacy	196 (67.6)
Independent Community Pharmacy	94 (32.4)
**Average Number of Total Patients You Serve Per Day**	
<10	38 (13.1)
10–50	190 (65.5)
51–100	55 (19)
>100	7 (2.4)
**Received Training Regarding Asthma**	
Yes	37 (12.8)
No	253 (87.2)
**Interested in Receiving Asthma Specific Training**	
Yes	142 (49)
No	148 (51)

**Table 2:** Knowledge of community pharmacists about bronchial asthma (N = 290).

Knowledge Domain	Knowledge Item	Correct Answer Frequency (%)
Asthma symptom recognition	Frequent wheezing or coughing (especially at night or in the early morning) are signs of asthma	236 (90.7)
Asthma triggers	Asthma can be triggered by different factors (e.g., Sinus infections and allergies)	246 (84.8)
Asthma epidemiology and disease characteristics	The majority of patients with asthma are cigarette smokers	157 (54.1)
Asthma control assessment	A patient who requires reliever medications more than twice/week, his/her asthma is considered well-controlled	173 (59.7)
Guideline-based asthma management (GINA)	Latest GINA guidelines recommend using long-acting beta2-agonist and inhaled corticosteroids as first choice reliever of symptoms in asthma adults	176 (60.7)
	Latest GINA guidelines no longer recommend short-acting beta2 agonist (SABA)-only therapy as it raises the danger of life-threatening exacerbations and deaths due to asthma	169 (58.3)
Appropriateness of pharmacotherapy	It is appropriate to use cough suppressants to treat coughing caused by asthma	186 (64.1)
Determinants of poor asthma control	Factors like poor inhaler technique and non-adherence lead to poor asthma control	263 (90.7)
Medication-related adverse effects	Inhaler medications containing salbutamol could cause oral candidiasis	171 (59)
Counseling on prevention of medication adverse effects	To reduce the risk of oral candidiasis, patients need to rinse their mouth with water and spit out after using inhaled corticosteroids or use spacers	273 (94.1)

**Table 3:** Practices of community pharmacists towards asthma (N = 290).

Practice	Always n%	Often n%	Sometimes n%	Rarely n%	Never n%	Median (25–75 Percentile)
I educate patients about basic facts of asthma	43 (14.8)	160 (55.2)	73 (25.2)	12 (4.1)	2 (0.7)	4 (3–4)
I can identify and manage modifiable risk factors (allergens, smoking, …) for poor asthma outcomes for the patient	54 (18.6)	124 (42.8)	98 (33.8)	11 (3.8)	3 (1)	4 (3–4)
I evaluate symptom control for the patient over the last 4 weeks	18 (6.2)	82 (28.3)	121 (41.7)	59 (20.3)	10 (3.4)	3 (3–4)
I evaluate asthmatic patients regarding their adherence to their prescribed regimens	44 (15.2)	98 (33.8)	111 (38.3)	26 (9)	11 (3.8)	3 (3–4)
I ask asthmatic patients about their asthma treatment preferences	68 (23.4)	72 (24.8)	110 (37.9)	32 (11)	8 (2.8)	3 (3–4)
I ask asthmatic patients about any treatment side effects they may have	52 (17.9)	96 (33.1)	98 (33.8)	35 (12.1)	9 (3.1)	4 (3–4)
I teach asthmatic patients how to self-monitor their symptoms	47 (16.2)	82 (28.3)	107 (36.9)	43 (14.8)	11 (3.8)	3 (3–4)
I educate asthmatic patients about their written asthma action plan	37 (12.8)	80 (27.6)	86 (29.7)	66 (22.8)	21 (7.2)	3 (2–4)
I educate patients on medication information (indications, directions, monitoring, side effects)	48 (16.6)	99 (34.1)	95 (32.8)	32 (11)	16 (5.5)	4 (3–4)
I educate patients about the proper technique of inhaled medications	52 (17.9)	80 (27.6)	85 (29.3)	50 (17.2)	23 (7.9)	3 (2–4)
I advise asthmatic patients to consult me before discontinuing any medication	18 (6.2)	40 (13.8)	118 (40.7)	83 (28.6)	31 (10.7)	3 (2–3)
I communicate with physicians regarding any drug-related problems and/or patient concerns	25 (8.6)	68 (23.4)	77 (26.6)	82 (28.3)	38 (13.1)	3 (2–4)
I schedule a follow-up visit for asthma patients	10 (3.4)	40 (13.8)	73 (25.2)	114 (39.3)	53 (18.3)	2 (2–3)

**Table 4:** Barriers to providing asthma management counseling (N = 290).

Barrier	n (%)
I do not have enough time	176 (60.7)
Patients do not have time	183 (63.1)
Patient’s perception that it is not the pharmacist’s role	155 (53.4)
Lack of asthma training programs for pharmacists	129 (44.5)
No financial incentive	126 (43.4)
I do not have enough knowledge	100 (34.5)
Cultural or religious barriers	90 (31)
No access to reliable resources/national or international guidelines	68 (23.4)
I do not have/cannot provide an asthma action plan	56 (19.3)
I do not think that it is my responsibility to counsel patients to improve asthma control	49 (16.9)

**Table 5:** Multivariable binary logistic regression analysis of factors associated with high asthma counseling practice among community pharmacists.

Predictor	Adjusted OR	95% CI	*p*-Value
High knowledge (yes vs. no)	2.34	1.35–4.06	0.002
Interested in asthma training (yes vs. no)	2.61	1.50–4.55	<0.001
Received asthma training (yes vs. no)	1.09	0.48–2.48	0.839
Patients served per day	1.57	0.998–2.47	0.051
Pharmacy type (independent vs. chain)	0.97	0.52–1.79	0.910
Location of pharmacy	-	-	0.257
Work experience	1.54	1.11–2.14	0.01
Country awarding degree (Saudi vs. outside)	0.82	0.41–1.65	0.584
Gender (female vs. male)	1.15	0.6–2.2	0.674

OR: odds ratio; CI: confidence interval. Multivariable binary logistic regression analysis was performed to identify predictors of high asthma counseling practice among community pharmacists (n = 290). The dependent variable was counseling practice, categorized as high vs. low based on the median score. All sociodemographic and professional variables shown in the table were entered simultaneously into the regression model using the ENTER method. Results are presented as adjusted odds ratios with 95% confidence intervals. Variables with ordered categories (e.g., years of work experience and number of patients served per day) were treated as ordinal predictors and presented as a single odds ratio per category increase. For variables with multiple categories (e.g., pharmacy location), only the overall *p*-value is reported. Reference categories were low knowledge, no asthma training, chain pharmacy, male gender, and non-Saudi degree. Model summary: n = 290; −2 Log Likelihood = 349.563; Model χ^2^ = 52.118, *p* < 0.001; Nagelkerke R^2^ = 0.219.

**Table 6:** Multivariable binary logistic regression analysis of factors associated with high asthma knowledge among community pharmacists.

Predictor	Adjusted OR	95% CI	*p*-Value
High practice (yes vs. no)	2.38	1.36–4.16	0.002
Interested in asthma training (yes vs. no)	1.25	0.7–2.2	0.452
Received asthma training (yes vs. no)	1.02	0.45–2.33	0.959
Patients served per day	1.07	0.67–1.69	0.784
Pharmacy type (independent vs. chain)	2.99	1.57–5.69	<0.001
Location of pharmacy	-	-	0.034
Work experience	1.8	0.84–1.64	0.342
Country awarding degree (Saudi vs. outside)	0.58	0.28–1.19	0.134
Gender (female vs. male)	1.15	0.58–2.3	0.687

OR: odds ratio; CI: confidence interval. Multivariable binary logistic regression analysis was performed to identify predictors of high asthma knowledge among community pharmacists (n = 290). The dependent variable was knowledge categorized as high vs. low based on the median score. All sociodemographic and professional variables shown in the table were entered simultaneously into the regression model using the ENTER method. Results are presented as adjusted odds ratios with 95% confidence intervals. Variables with ordered categories (e.g., years of work experience and number of patients served per day) were treated as ordinal predictors and presented as a single odds ratio per category increase. For variables with multiple categories (e.g., pharmacy location), only the overall *p*-value is reported. Reference categories were low practice, no asthma training, chain pharmacy, male gender, and non-Saudi degree. Model summary: n = 290; −2 Log Likelihood = 340.413; Model χ^2^ = 35.722, *p* < 0.001; Nagelkerke R^2^ = 0.159.

## Data Availability

The raw data supporting the conclusions of this article will be made available by the authors on request.
